# Contribution of *TEX15* genetic variants to the risk of developing severe non-obstructive oligozoospermia

**DOI:** 10.3389/fcell.2022.1089782

**Published:** 2022-12-15

**Authors:** Andrea Guzmán-Jiménez, Sara González-Muñoz, Miriam Cerván-Martín, Rocío Rivera-Egea, Nicolás Garrido, Saturnino Luján, Samuel Santos-Ribeiro, José A. Castilla, M. Carmen Gonzalvo, Ana Clavero, F. Javier Vicente, Vicente Maldonado, Javier Villegas-Salmerón, Miguel Burgos, Rafael Jiménez, Maria Graça Pinto, Isabel Pereira, Joaquim Nunes, Josvany Sánchez-Curbelo, Olga López-Rodrigo, Iris Pereira-Caetano, Patricia Isabel Marques, Filipa Carvalho, Alberto Barros, Lluís Bassas, Susana Seixas, João Gonçalves, Alexandra M. Lopes, Sara Larriba, Rogelio J. Palomino-Morales, F. David Carmona, Lara Bossini-Castillo, IVIRMA Group, Lisbon Clinical Group

**Affiliations:** ^1^ Departamento de Genética e Instituto de Biotecnología, Centro de Investigación Biomédica (CIBM), Universidad de Granada, Granada, Spain; ^2^ Instituto de Investigación Biosanitaria ibs.GRANADA, Granada, Spain; ^3^ Andrology Laboratory and Sperm Bank, IVIRMA Valencia, Valencia, Spain; ^4^ IVI Foundation, Health Research Institute La Fe, Valencia, Spain; ^5^ Servicio de Urología. Hospital Universitari i Politecnic La Fe e Instituto de Investigación Sanitaria La Fe (IIS La Fe), Valencia, Spain; ^6^ IVI-RMA Lisbon, Lisbon, Portugal; ^7^ Department of Obstetrics and Gynecology, Faculty of Medicine, University of Lisbon, Lisbon, Portugal; ^8^ Unidad de Reproducción, UGC Obstetricia y Ginecología, HU Virgen de Las Nieves, Granada, Spain; ^9^ CEIFER Biobanco—GAMETIA, Granada, Spain; ^10^ UGC de Urología, HU Virgen de las Nieves, Granada, Spain; ^11^ UGC de Obstetricia y Ginecología, Complejo Hospitalario de Jaén, Jaén, Spain; ^12^ Centro de Medicina Reprodutiva, Maternidade Alfredo da Costa, Centro Hospitalar Universitário de Lisboa Central, Lisboa, Portugal; ^13^ Departamento de Obstetrícia, Ginecologia e Medicina da Reprodução, Hospital de Santa Maria, Centro Hospitalar Universitário de Lisboa Norte, Lisboa, Portugal; ^14^ Laboratory of Seminology and Embryology, Andrology Service-Fundació Puigvert, Barcelona, Spain; ^15^ Departamento de Genética Humana, Instituto Nacional de Saúde Dr. Ricardo Jorge, Lisbon, Portugal; ^16^ i3S—Instituto de Investigação e Inovação em Saúde, Universidade do Porto, Porto, Portugal; ^17^ Institute of Molecular Pathology and Immunology of the University of Porto (IPATIMUP), Porto, Portugal; ^18^ Serviço de Genética, Departamento de Patologia, Faculdade de Medicina da Universidade do Porto, Porto, Portugal; ^19^ ToxOmics—Centro de Toxicogenómica e Saúde Humana, Nova Medical School, Lisbon, Portugal; ^20^ CGPP-IBMC—Centro de Genética Preditiva e Preventiva, Instituto de Biologia Molecular e Celular, Universidade do Porto, Porto, Portugal; ^21^ Human Molecular Genetics Group, Bellvitge Biomedical Research Institute (IDIBELL), L’Hospitalet de Llobregat, Barcelona, Spain; ^22^ Departamento de Bioquímica y Biología Molecular I, Universidad de Granada, Granada, Spain

**Keywords:** oligozoospermia, spermatogenesis, TEX15, polymorphisms, association study

## Abstract

**Background:** Severe spermatogenic failure (SPGF) represents one of the most relevant causes of male infertility. This pathological condition can lead to extreme abnormalities in the seminal sperm count, such as severe oligozoospermia (SO) or non-obstructive azoospermia (NOA). Most cases of SPGF have an unknown aetiology, and it is known that this idiopathic form of male infertility represents a complex condition. In this study, we aimed to evaluate whether common genetic variation in *TEX15*, which encodes a key player in spermatogenesis, is involved in the susceptibility to idiopathic SPGF.

**Materials and Methods:** We designed a genetic association study comprising a total of 727 SPGF cases (including 527 NOA and 200 SO) and 1,058 unaffected men from the Iberian Peninsula. Following a tagging strategy, three tag single-nucleotide polymorphisms (SNPs) of *TEX15* (rs1362912, rs323342, and rs323346) were selected for genotyping using TaqMan probes. Case-control association tests were then performed by logistic regression models. *In silico* analyses were also carried out to shed light into the putative functional implications of the studied variants.

**Results:** A significant increase in *TEX15*-rs1362912 minor allele frequency (MAF) was observed in the group of SO patients (MAF = 0.0842) compared to either the control cohort (MAF = 0.0468, OR = 1.90, *p* = 7.47E-03) or the NOA group (MAF = 0.0472, OR = 1.83, *p* = 1.23E-02). The genotype distribution of the SO population was also different from those of both control (*p* = 1.14E-02) and NOA groups (*p* = 4.33–02). The analysis of functional annotations of the human genome suggested that the effect of the SO-associated *TEX15* variants is likely exerted by alteration of the binding affinity of crucial transcription factors for spermatogenesis.

**Conclusion:** Our results suggest that common variation in *TEX15* is involved in the genetic predisposition to SO, thus supporting the notion of idiopathic SPGF as a complex trait.

## Introduction

Infertility is a growing health concern involving over 50–70 million childbearing age couples worldwide, with the male factor contributing in approximately 50% of cases (Fainberg and Kashanian, 2019). Non-obstructive azoospermia (NOA) and severe oligozoospermia (SO) due to spermatogenic failure (SPGF) represent the most severe phenotypes of male factor infertility. However, the causes of such conditions are poorly understood and the aetiology of most affected men is usually defined as being idiopathic (Cannarella et al., 2019; Agarwal et al., 2021). Recent advances clearly point to idiopathic male infertility as a complex trait, in which the combined effect of polymorphic risk variants (causing subtle changes in gene expression) may increase the susceptibility of an individual to suffer from this disorder (Singh and Jaiswal, 2011; Cervan-Martin et al., 2020b; Salas-Huetos and Aston, 2021).

In this regard, common variants in the human genome may increase the susceptibility to develop male infertility by altering key events during spermatogenesis, in which DNA integrity is crucial (Neto et al., 2016). Having such in mind, it is important to stress that alterations in the DNA structure frequently occur in meiosis prophase I during the transition from primary to secondary spermatocytes, due to the generation of double-strand breaks (DSBs) for homologous chromosome pairing and crossing over (CO) (Cannarella et al., 2020). Consequently, DNA repair mechanisms are essential to produce fit mature sperm cells. In fact, the assessment of DNA integrity is a relevant marker to determine the sperm quality in assisted reproductive techniques (ART) (Leduc et al., 2008; Gonzalez-Marin et al., 2012). Thus, while the generation of genetic variability through changes in DNA is the basis for evolution, it is strictly controlled at the individual level (Capilla et al., 2016; Alves et al., 2017).

Consistent with the above, the spermatogenic process shows a very complex molecular and cellular control, involving over 2,000 genes, among which 900 are solely expressed in the male germline (Chalmel et al., 2012; Cervan-Martin et al., 2020b). The so-called *Testis expressed* (TEX) gene family plays a crucial role in spermatogenesis and some of its members have been strongly associated with male infertility (Cannarella et al., 2019; Bellil et al., 2021). Specifically, *TEX15*, which is predominantly expressed in spermatogonial and primary spermatocytes, encodes a 2,789 amino acid protein necessary for meiotic recombination and DSB repair in the germ line (Yang et al., 2008). Additionally, TEX15 also has a significant epigenetic function in spermatogenesis by interacting with several PIWI like RNA-mediated gene silencing proteins (namely PIWIL2, PIWIL4), in order to maintain spermatogonial stem cell integrity through the silencing of transposable elements (Schopp et al., 2020; Yang et al., 2020). Indeed, inactivation of *Tex15* expression in mutant male mice causes sterility by meiotic arrest and impaired activity of *RAD51* and *DMC1* recombinases (Yang et al., 2008; Chen et al., 2016).

In this context, high-penetrance pathogenic variants in *TEX15* have been widely linked to human male infertility due to spermatogenic impairment in different studies. For instance, non-sense mutations producing truncated TEX15 proteins have been reported to lead to cryptozoospermia (where spermatozoa are apparently absent in fresh semen samples but are recovered in centrifuged pellets) and SPGF in unrelated families (Okutman et al., 2015; Colombo et al., 2017; Wang et al., 2018; Cannarella et al., 2021; Tian et al., 2021). Moreover, mutations in this gene have been associated with NOA due to Sertoli cell-only (SCO) phenotype (characterised by a complete lack of germ cells in the seminiferous tubules) (Araujo et al., 2020).

On the other hand, case-control genetic association studies have attempted to assess the involvement of *TEX15* polymorphic variation, such as single nucleotide polymorphisms (SNPs), in the development of SPGF resulting in NOA or SO. The first approach was conducted in a cohort of European ancestry and identified two *TEX15* SNPs putatively associated with SPGF (Aston et al., 2010). However, such associations were not replicated in a subsequent study carried out in an independent South-Eastern European population (Plaseski et al., 2012). Similarly, two novel *TEX15* variants have been recently proposed to be involved in the genetic predisposition to SPGF in the Iranian population (Ghadirkhomi et al., 2022). Finally, although *TEX15* common variation was initially associated with SPGF risk in Han Chinese (Ruan et al., 2012), another report could not confirm this observation in this ethnicity (Zhang et al., 2015). Therefore, no conclusive results are currently available on the possible role of the common *TEX15* genetic variation in SPGF development.

In view of the above, we aimed to clarify the involvement of *TEX15* common variation in SPGF predisposition in a European genetic background, by analysing a large and phenotypically well-characterised study cohort.

## Materials and methods

### Patient cohort characterisation

This study was carried out in a large SPGF cohort with European descent. In total, the infertile men group comprised 727 SPGF cases from Spain and Portugal, diagnosed as NOA (*n* = 527), if a total absence of spermatozoa was observed in the ejaculate, or SO (*n* = 200), when showing less than 5 million spermatozoa/mL semen. The control set included 1,058 Iberian men, of whom 700 were healthy individuals representative of the general population (most of them with self-reported fatherhood), and 358 men with normal sperm counts, as previously described (Cervan-Martin et al., 2020a; Cervan-Martin et al., 2020c). Cases and controls were matched by age, geographical origin, and ethnicity, and signed an informed written consent in accordance with the Declaration of Helsinki. Each participating centre received ethical approval and complied with the requirements of their local regulatory authorities prior to the study.

SPGF patients were diagnosed after two high-speed centrifugations in two different semen samples in different fertility clinics managed in public and private health Hospitals and centres from Portugal and Spain, based on the guidelines for the management of infertile men by the American Urological Association (AUA)/American Society for Reproductive Medicine (ASRM), the Canadian Urological Association (CUA), and the World Health Organization (WHO) (Cooper et al., 2010; Jarvi et al., 2010; Bjorndahl and Kirkman Brown, 2022). Moreover, the NOA diagnosis was further confirmed by histological examination of testicular biopsies of the NOA patients that decided to undergo testicular sperm extraction (TESE) for use in ART (who represented around half of the NOA cohort). We established stringent selection criteria to include only infertile men due to idiopathic SPGF, which involved an exhaustive medical evaluation, screening for possible karyotype abnormalities and Y-chromosome microdeletions, as well as a thorough revision of the medical records to discard physical testicular disorders (e.g., orchitis and obstruction of vas deferens) and other known causes of male infertility. However, no screening for high-penetrance point mutations was conducted because this procedure is not part of the routine diagnostic workup of infertile men due to SPGF (Krausz et al., 2018).

### Study design and single nucleotide polymorphism selection

A candidate gene study was conducted to shed light on the possible association of common genetic variation in the *TEX15 locus* with idiopathic SPGF risk. During the selection process, we confirmed that, according to publicly available single-cell RNA-seq data in the puberty and adult (Guo et al., 2018; Guo et al., 2020), the highest *TEX15* expression was found in spermatogonia and spermatocytes (Supplementary Figure S1).

**FIGURE 1:**
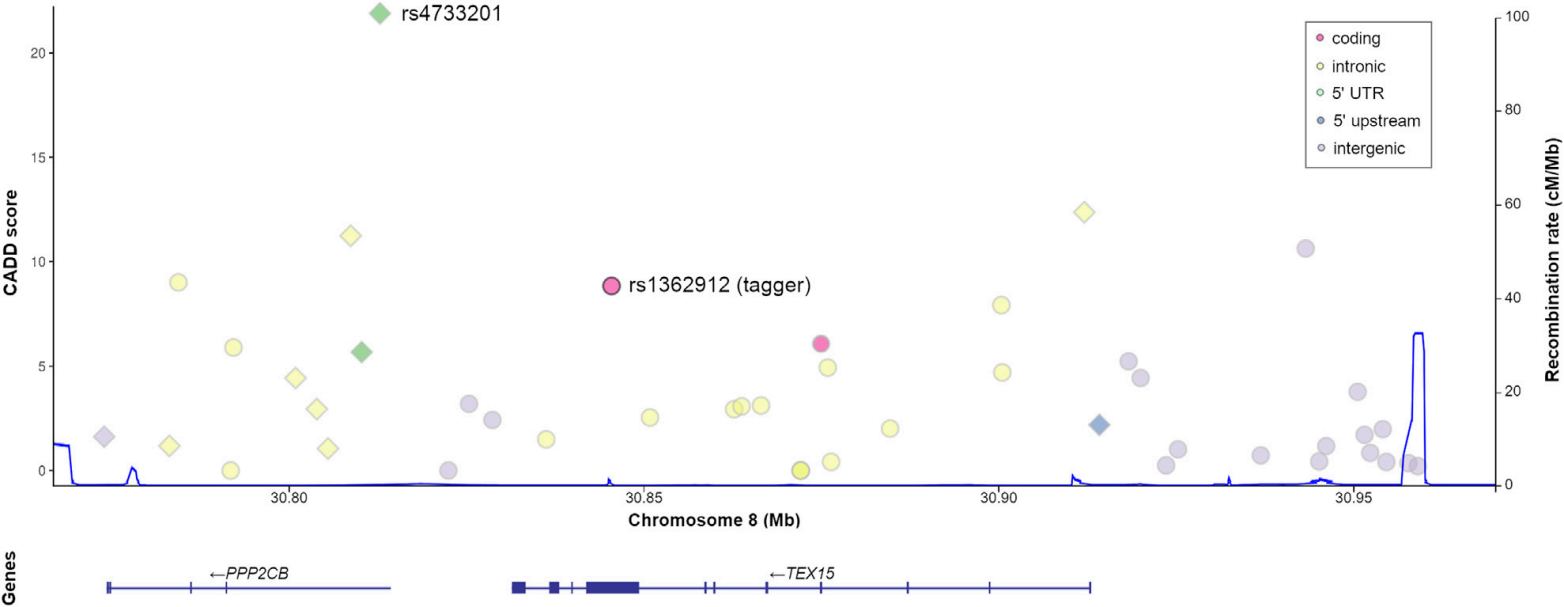
Genomic context and predicted functional relevance of the proxy variants tagged by *TEX15*-rs1362912. Both the CADD scores of each variant (left *x*-axis) and the recombination rate of the region (right *x* axis, blue line) are displayed. Variants with functional annotations in testis are represented as diamonds.

*TEX15* is located in human chromosome 8, spanning an 81.5 kb region that constitutes a single linkage disequilibrium (LD) block in the 1,000 Genomes phase III (1KGPh3) European population (Auton et al., 2015), according to LDlink (Machiela and Chanock, 2015) (Figure 1A). Therefore, we followed a SNP tagging strategy using the European data of the 1KGPh3 and the Haploview V.4.2 software (Barrett, 2009; Auton et al., 2015) to cover most of the genetic variability of the region, prioritising those taggers that tagged, at least, more than five variants. Three taggers representative of three different minor allele frequency (MAF) ranges were selected with this method: rs1362912 (MAF <0.1), and rs323346 (0.1 < MAF <0.2), and rs323342 (MAF >0.2) (Supplementary Table S1).

### Sample preparation and genotyping

For all the recruited individuals, genomic DNA was extracted from peripheral blood mononuclear cells with the QIAamp^®^ DNA Blood Midi/Maxi kit (Qiagen, Hilden, Germany), the MagNA Pure LC-DNA LV Isolation kit I (Roche, Basel, Switzerland), or the Wizard^®^ Genomic DNA Purification Kit Protocol (Promega, Madison, WI, United States), following the manufacturers’ protocols. The genotyping was performed using the TaqMan™ SNP genotyping technology (Applied Biosystems, Foster City, CA, United States). Real-time quantitative polymerase chain reactions (qPCR) were performed in a 7900HT Fast Real-Time PCR System (Applied Biosystems, Foster City, California, United States), using specific predesigned TaqMan™ probes (assay IDs: C___8867446_10; C____622151_10; C____622153_10) and the SDS 2.3 software for allele discrimination (both from Applied Biosystems, Foster City, California, United States).

### Case-control statistical association analysis

An estimation of the statistical power of this study was calculated with the CaTS Power Calculator for Genetic Association Studies (Skol et al., 2006) (Supplementary Table S2). Possible deviances from Hardy-Weinberg equilibrium (HWE) were evaluated at the 5% significance level in both case and control groups using a χ^2^ test.

The statistical analyses were conducted with the software Plink v1.9 (Chang et al., 2015). Case-control comparisons of the allele and genotype frequencies were performed by logistic regression on the genotypes using geographical origin (Spain or Portugal) as a covariate and assuming different association models for genetic risk (including additive, dominant, recessive, and genotypic). *p*-values, odds ratios (ORs) and their 95% confidence intervals (CIs) were then calculated setting the significance threshold at *p*-value < 0.05 after correction of possible multiple testing effects by the Benjamini and Hochberg False Discovery Rate (FDR-BH) method (Benjamini et al., 2001).

### *In silico* functional characterisation of risk variants

In an attempt to provide a plausible functional effect of the observed associations and identify possible molecular or cellular mechanisms underlying the pathogenic phenotypes, we implemented a bioinformatic workflow to extract and explore the functional annotation data available in different public databases. In a first step, we extended our functional characterisation to all proxies (genetic variants showing a LD *r* ^2^ ≥ 0.8) of the selected taggers in the reference European population using the tools for that purpose implemented in LDLink (Machiela and Chanock, 2015). All proxies were equally considered candidates for explaining the observed associations, as described elsewhere (Cervan-Martin et al., 2020a; Cervan-Martin et al., 2021). Briefly, the prioritisation processes were performed with the following resources: GTExPortal (Carithers and Moore, 2015), SNPnexus (Oscanoa et al., 2020), ENCODE (Luo et al., 2020), Haploreg v.4.1. (Ward and Kellis, 2016), RegulomeDB (Dong and Boyle, 2019), SNP2TFBS (Kumar et al., 2017), amongst others. Several scores of deleteriousness were also used, such as CADD, DeepSEA, EIGEN, FATHMM, fitCons, FunSeq2 GWAVA, REMM, and RegulomeDB (Supplementary Tables S3,S4). In addition, we carried out an enrichment analysis of both gene ontology (GO) terms and protein-protein interactions (PPIs), including all transcription factors whose reported binding site (TFBS) sequences overlapped with the SNPs included in the prioritisation analysis, as implemented in STRINGv11.5 (Szklarczyk et al., 2019). Finally, to determine the *TEX15* expression at the single cell level, we used the data available at both the Human Testis Atlas Browser (Guo et al., 2018) and the Single Cell Expression Atlas (Papatheodorou et al., 2020).

## Results

The genotyping success rate of the three analysed variants was above 98% and none of them showed a significant deviation from HWE either in cases or controls. Additionally, the MAFs in the control group were concordant with those described for both the Iberian subpopulation and the European super population of the 1KGPh3 project (Auton et al., 2015), with no statistically significant differences observed between the population-representative group and the normozoospermic group in either the allele or the genotype frequencies. Furthermore, our study population had an appropriate overall statistical power to identify genetic associations with moderate to high effects, as detailed in Supplementary Table S2.

### Common *TEX15* gene variation confers susceptibility to severe oligozoospermia

First, we compared the allele and genotype frequencies of the selected taggers between the overall SPFG group and the unaffected control population. These analyses revealed no significant differences under any of the tested models (Table 1).

**TABLE 1:** Analysis of the genotype and allele frequencies of the *TEX15* tagger variants comparing groups of male infertility against the unaffected control group.

					Allelic model			Genotypic model	
SNP	Change (1/2)	Group	Genotypes (11/12/22)	MAF	*p*	Adjusted *p*	OR [CI 95%]	*p*	Adjusted *p*
rs1362912	G/A	Controls (*n* = 1,046)	1/96/949	0.0468	NA	NA	NA	NA	NA
		SPGF (*n* = 715)	4/74/637	0.0573	0.2571	NS	1.20 [0.88–1.65]	0.2367	NS
		SO (*n* = 196)	2/29/165	0.0842	**7.47E-03**	**2.24E-02**	1.90 [1.19–3.03]	**1.14E-02**	**3.41E-02**
		NOA (*n* = 519)	2/45/472	0.0472	0.9884	NS	1.00 [0.70–1.43]	0.4932	NS
rs323342	A/T	Controls (*n* = 1,049)	67/420/562	0.2641	NA	NA	NA	NA	NA
		SPGF (*n* = 714)	60/277/377	0.2780	0.6212	NS	1.04 [0.89–1.22]	0.5412	NS
		SO (*n* = 195)	18/67/110	0.2641	0.6740	NS	0.94 [0.72–1.23]	0.3335	NS
		NOA (*n* = 519)	42/210/267	0.2832	0.4275	NS	1.07 [0.90–1.27]	0.6689	NS
rs323346	C/T	Controls (*n* = 1,049)	32/318/699	0.1821	NA	NA	NA	NA	NA
		SPGF (*n* = 718)	29/225/464	0.1971	0.5822	NS	1.05 [0.88–1.26]	0.7808	NS
		SO (*n* = 197)	7/64/126	0.1980	0.7353	NS	1.05 [0.78–1.43]	0.9444	NS
		NOA (*n* = 521)	22/161/338	0.1967	0.5371	NS	1.06 [0.88–1.29]	0.6949	NS

CI, confidence interval; MAF, minor allele frequency; NA, not applicable; NOA, non-obstructive azoospermia; NS: not significant; OR, odds ratio (for the minor allele); SNP, single-nucleotide polymorphism; SO, severe oligozoospermia; SPGF, spermatogenic failure. Significant *p*-values are highlighted in bold. 1: reference (minor) allele. 2: alternative (major) allele.

Subsequently, we compared the groups of cases showing specific phenotypes of male infertility stablished by semen analysis (that is, NOA and SO) against the control cohort. Regarding NOA, no statistically significant differences were observed for any of the three tested taggers. In fact, the MAFs for the NOA group were very similar to those observed for the control group (Table 1).

Conversely, the comparison between the SO group and the control cohort revealed statistically significant differences in the allele/genotype frequencies of rs1362912 under the additive (*p*_ADD_ = 7.47E-03, OR = 1.90, CI 95% = 1.19–3.03) and genotypic (*p*_GENO_ = 1.14E-02) models (Table 1). Such associations remained significant after multiple testing correction (*p*_ADD-FDR_ = 2.24E-02; *p*_GENO-FDR_ = 3.41E-02) (Table 1).

To assess the consistency of the rs1362912 association with SO, we decided to eliminate the possible confounding effect of having SPFG in the analysis by using the NOA group as the reference cohort. The comparison between SO and NOA showed a similar risk effect on SO for the rs1362912*G minor allele in the additive model (*p*_ADD_ = 1.23E-02, 1.83, CI 95% = 1.14–2.94). The observed differences in the genotypic test for this SNP were also at the same range (*p*_GENO_ = 4.33E-02) (Table 2). Interestingly, despite the lower statistical power of this case-case comparison, the association assuming an additive effect of rs1362912*G rameined after multiple testing correction (*p*_ADD-FDR_ = 3.69E-02) (Table 2).

**TABLE 2:** Analysis of the genotype and allele frequencies of the *TEX15* tagger variants comparing the severe oligozoospermia (SO) group against the non-obstructive azoospermia (NOA) group.

		Allelic model			Genotypic model	
SNP	MAF (SO/NOA)	*p*	Adjusted *p*	OR [CI 95%]^	*p*	Adjusted *p*
rs1362912	0.0842/0.0472	**1.23E-02**	**3.69E-02**	1.83 [1.14–2.94]	**4.33E-02**	0.13
rs323342	0.2641/0.2832	0.3614	NS	0.88 [0.67–1.15]	0.2672	NS
rs323346	0.1980/0.1967	0.6516	NS	0.93 [0.69–1.26]	0.7451	NS

CI, confidence interval; MAF, minor allele frequency; NS: not significant; OR, odds ratio (for the minor allele); SNP, single nucleotide polymorphism; Significant *p*-values are highlighted in bold.

No evidence of additional association with the SO group was evident for the remaining selected variants (i.e., rs323346 and rs323342) (Tables 1, 2).

### The severe oligozoospermia-associated variants in *TEX15* might affect chromatin activity in the testis

The statistical analyses suggested an involvement of low frequency variants, tagged by rs1362912, in the susceptibility to SO. However, our study design relied on a tagging strategy, and the studied SNPs were selected based on their representativeness of different MAFs rather than on their possible functional features. Therefore, to investigate the biological meaning of the observed association between rs1362912 and SO further, we first identified all proxies (*r* ^2^ ≥ 0.8) of this tagger and, subsequently, we carried out a functional prioritisation to elucidate the putative causal variant/s.

**FIGURE 2:**
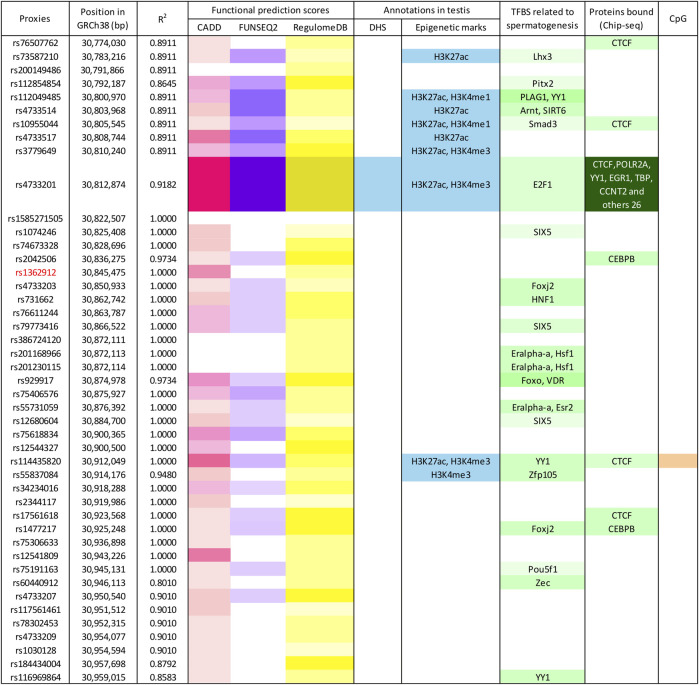
Enrichment in functional annotations of the human genome for *TEX15*-rs1362912 and its proxies. Functional prediction scores, according to different algorithms, are marked in pink (CADD), violet (Funseq2), and yellow (RegulomeDB); blue-coloured cells represent overlap with functional annotations in testis based on the ENCODE and Roadmap Epigenomics projects; variants located within transcription factor binding sites (TFBS) related to spermatogenesis (based on either position weight matrix data or Chip-seq experiments) are shown in green; and variants in CpG sites are highlighted in orange. Colour intensity is correlated with the probability or relevance (dark colours indicate higher probability). Bp, base pairs; GRCh38, Genome Reference Consortium Human Build 38; DHS, DNase I hypersensitive site.

The tagger rs1362912 represents a synonymous SNP in exon eight of the *TEX15* gene (Figure 1), whereas most of its *proxies* are non-coding variants located in both intronic and intergenic regions (Figure 1; Supplementary Table S5). We decided to base our prioritisation mostly on the overlaps with possible regulatory elements in the testis related with changes in gene expression that could affect the spermatogenic process. Amongst the 44 SNPs that were evaluated, the variants rs114435820, rs4733201, and rs1381559038 stood out from the rest using such criterion (Figure 2; Supplementary Table S5). Specifically, the SNP rs114435820 was located in a CpG island, which may indicate a possible effect on nearby gene expression. Interestingly, this proxy overlaps with a CTCF binding site, as well as with histone marks to active enhancers (H3K27ac) and promoters (H3K4me3) in the adult testis, according to ChIP- seq data from ENCODE (Davis et al., 2018) (Figure 2; Supplementary Table S5). Additionally, the position weight matrix (PWM) data extracted from HaploReg (Ward and Kellis, 2016) showed that the SNP rs114435820 could alter the binding affinity of the transcription factor YY1, which plays an important role in DNA repair during spermatogenesis (Wu et al., 2009), amongst others (Figure 2; Supplementary Tables S5, S6). The data of the testis from ENCODE also showed an overlap of the variant rs4733201 with binding sites of CTCF and POLR2A, with the same previously described histone marks, and with a DNAse hypersensitive sites (Figure 2; Supplementary Table S5). Furthermore, this variant was predicted to alter the TFBS of E2F1, which has been previously associated with male infertility due to SPGF (Jorgez et al., 2015; Rocca et al., 2019). Moreover, 31 additional proteins were observed to be bound to this site through ChIP-Seq experiments, being some of them (such as CTCF, YY1, EGR1, TBP, or CCNT2) involved in spermatogenesis (Tourtellotte et al., 2000; Wu et al., 2009; Teng et al., 2011; Hernandez-Hernandez et al., 2016; Ponomarenko et al., 2020) (Figure 2; Supplementary Tables S5, S6). With regards to the scores indicative of functionality, although some *proxies* (such as rs114433201) showed relevant values, the strongest evidence of deleteriousness was observed for the *TEX15* variant rs4733201, which is located in the 5’ untranslated region of the nearby gene *PPP2CB* and had a considerably high CADD score (score = 21.9) (Figures 1, 2; Supplementary Table S5).

**FIGURE 3:**
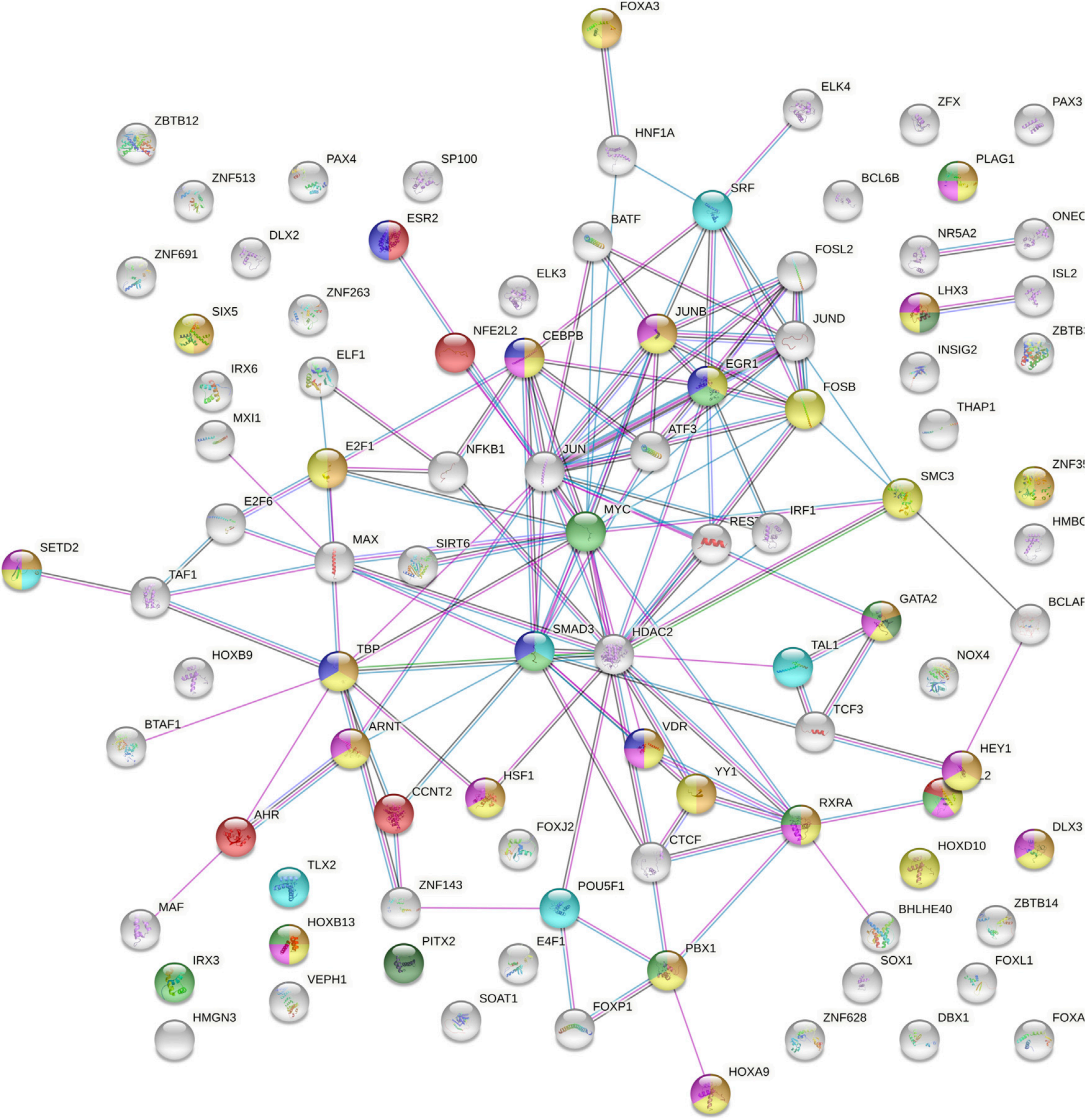
Protein-protein interaction network of the 122 transcription factors with predicted binding sites overlapping with *TEX15*-rs1362912 and its proxies based on ChIP-seq experiments or protein weight matrix data. Blue lines indicate that the interaction is established from curated databases; pink lines indicate experimentally determined connections; green lines link neighbour genes; black lines represent co-expression; and grey lines correspond to proteins with homology. Bubble colour meaning: light blue, formation of primary germ layer; orange, developmental process involved in reproduction; yellow, reproductive process; pink, reproductive structure development; dark green, pituitary gland development; light green, urogenital system development; purple, ovarian infertility and red, male infertility.

In view of the possible influence on TFBSs of the most likely causal variants of the *TEX15* association with SO, we decided to perform an enrichment analysis of both PPIs and biological pathways considering the 112 TFs whose binding affinity to this genetic region was predicted to be altered by the analysed SNPs (Supplementary Tables S5, S6). The PPI network showed significantly more interactions than expected (*p* = 1.00E-16) (Figure 3). In relation to the functional enrichment of this protein set, “regulation of transcription” and “gene expression” were the GO terms showing the most significant enrichment *p*-values (*p* = 3.94E-61 and *p* = 2.60E-45, respectively). Interestingly, other significantly enriched biological pathways included “reproductive process” (GO:0022414; *p* = 2.98E-07), “developmental process involved in reproduction” (GO:0003006; *p* = 1.31E-07), “reproductive structure development” (GO:0048608; *p* = 3.51E-07), “urogenital system development” (GO:0001655; *p* = 2.8E-04), “formation of primary germ layer” (GO:0001704; *p* = 1.70E-03), and “pituitary gland development” (GO:0021983; *p* = 4.26E-02) (Figure 3; Supplementary Table S7). Furthermore, “male infertility” (WP4673; *p* = 9.00E-03) and “ovarian infertility” (WP34; *p* = 4.48E-06) were also highlighted amongst the enriched pathways (Figure 3; Supplementary Table S8).

## Discussion

Understanding the genetic basis of SPGF is a necessary step to improve the clinical management and genetic counselling of infertile patients (Fainberg and Kashanian, 2019; Sharma et al., 2021). In the present study, we conducted a comprehensive evaluation of the possible involvement of *TEX15* genetic variation in the predisposition to SPGF, using a large European cohort that ensured a high statistical power.

Our results clearly suggest that *TEX15*-rs1362912, or any of its tagged SNPs, is directly involved in the pathological mechanisms underlying mild forms of SPGF, i.e., SO, with little or no contribution to extreme patterns, such as NOA. Taking into account the high relevance of *TEX15* in the reproductive function and the insight provided by previously published genetic studies on this gene (Aston et al., 2010; Plaseski et al., 2012; Ruan et al., 2012; Okutman et al., 2015; Zhang et al., 2015; Colombo et al., 2017; Wang et al., 2018; Araujo et al., 2020; Cannarella et al., 2021; Ghadirkhomi et al., 2022), we hypothesise that NOA could be mostly influenced by high-penetrance damaging mutations in this *locus* rather than by common SNPs, which would be responsible for increasing SO predisposition instead. Consistent with this idea, only 27 SO-associated point mutations are currently annotated in both the “Male Infertility Genomic Consortium (IMIGC) database” and the “Infertility Disease Database (IDDB)”, whereas NOA has 138 entries, amongst which *TEX15* is included (Houston et al., 2021; Wu et al., 2021). Moreover, a higher proportion of NOA cases present Mendelian causes of their infertility when compared to SO (e.g., AZF microdeletions and chromosomal abnormalities are three times more prevalent in NOA than in SO) (Krausz and Riera-Escamilla, 2018).

Although our data are consistent with this hypothesis, the causality of the *TEX15* common variation in SO predisposition remains controversial. Previous genetic association reports in this *locus* have not been fully replicated likely due to differences in the genetic architecture of the different study groups or, more probably, to type I or II errors caused by the limitations in the statistical power of the studied cohorts to detect low or modest effects. In this regard, the *TEX15* SNP rs323346 was not associated with SO neither in our study nor in an independent population of European ancestry previously assessed by Aston et al. (2010). Inconsistent results were obtained in two additional studies performed in Chinese, as Ruan et al. (2012) described a genetic association between this SNP and SO that was not replicated by Zhang et al. (2015) in the same ethnicity. Moreover, there are striking differences between the reported rs323346*C allele frequencies for the human populations included in the 1KGPh3 project (e.g., EUR = 0.17; EAS = 0.11; SAS = 0.33; AFR = 0.80, Supplementary Figure S2) (Auton et al., 2015), which might indicate population-specific causal variants within this region (Aston et al., 2010; Ruan et al., 2012; Ghadirkhomi et al., 2022). Therefore, subsequent studies in independent study cohorts should be performed to clarify further the possible existence of different SO functionally relevant SNPs in this gene.

Remarkably, animal models clearly support the role of the *TEX15 locus* in SO development (Yang et al., 2008; Schopp et al., 2020; Yang et al., 2020). In fact, *TEX15*-defficient murine models show reproductive system abnormalities, including male infertility, altered spermatogenesis, arrest of male meiosis, decreased male germ cell number, DSB repair anomalies, and reduced testis weight (Yang et al., 2008; Carvalho-Silva et al., 2019). This gene is mostly expressed in spermatogonia and spermatocytes, in which it has a relevant regulatory role in the control of gene expression by interacting with a vast number of transcription factors related to the reproductive function, as reported in the Open Targets Platform (Carvalho-Silva et al., 2019).

According to the results of our *in silico* functional analyses, amongst the linked polymorphisms of the tagger rs1362912, the non-coding *TEX15* variant rs4733201 may be responsible for an important contribution to the final phenotype. This SNP showed strong evidence of functionality, including high scoring by different functional impact prediction methods as well as an overlap with testis-specific active chromatin epigenetic marks. Interestingly, the sequence surrounding this SNP corresponds to a TFBS for different key transcription factors related with spermatogenesis, including EGR1 (Tourtellotte et al., 2000; Man et al., 2014), CTCF (Hernandez-Hernandez et al., 2016; Rivero-Hinojosa et al., 2021), SMC3 (Eijpe et al., 2000; James et al., 2002), YY1 (Wu et al., 2009; Kim et al., 2016), CCNT2 (Teng et al., 2011), E2F6 (Pohlers et al., 2005; Dahlet et al., 2021), and MAX (Maeda et al., 2013), and it is annotated to be transcriptionally active in the testis (Davis et al., 2018). Moreover, this SNP was predicted to modify the activity of E2F1, which has been associated with human SPGF (Jorgez et al., 2015; Rocca et al., 2019).

Similarly, the results of the biological pathway enrichment analysis of the transcription factors with TFBSs influenced by the analysed variants also support their involvement in SPGF, as some of the enriched pathways included “reproductive process”, “pituitary gland development”, “ovarian infertility”, and “male infertility”. To our knowledge, there are no reported studies in which the possible effects of *TEX15* on female infertility were evaluated (Yang et al., 2008; Bellil et al., 2021). However, *TEX15* is expressed in the female reproductive tract (Supplementary Figure S1), “ovarian infertility” was amongst the most enriched pathways, and there is evidence of the implications in female fertility of some of the transcription factors with binding sites in the genomic positions of some of the SNPs considered in our functional analysis. Specifically, VDR is associated with female infertility (Djurovic et al., 2020), whereas CEBPB has been described as relevant factor for female reproduction for its role in ovarian follicle development (Fan et al., 2009), together with SMAD3 (Tomic et al., 2004; Li et al., 2008) and ESR2 (Khristi et al., 2018; Chakravarthi et al., 2020). Such is consistent with the assumption that the effect of the common variation in the genome on pathological conditions is not limited to altering the expression or function of a single gene or protein, but rather to unbalance complex molecular networks (Cui et al., 2015).

Regarding the possible limitations of this work, it is important note that our exclusion criteria based on genetic abnormalities considered only karyotype alterations and Y chromosome microdeletions. Although both type of alterations account for the majority of known genetic causes of SPGF (Cioppi et al., 2021), screening of high-penetrance point mutations in reported SPGF genes was not performed. As a consequence, some cases of our study cohort could harbour single-gene mutations that may explain their infertility. However, considering the low frequency of the reported monogenic mutations in SPGF (Cioppi et al., 2021), this limitation is not expected to have a relevant impact in the allele frequencies of our study cohort. The possible statistical noise added by such cases would likely cause a subtle increase in the probability of obtaining type II errors (false negative results), but it would hardly affect the consistency of the observed associations.

Overall, the insight gained in this study supports the notion of idiopathic SPGF as a complex trait, where common genetic variation has a determinant role in disease susceptibility and development. In the case of *TEX15*, it is likely that the selective pressures have prevented the dissemination of high-penetrance deleterious variants leading to NOA in the human populations, considering the crucial role that this gene has in the reproductive success of an individual (Yang et al., 2008; Chen et al., 2016). Under this assumption, it is reasonable to assume that the common *TEX15* polymorphisms affecting male infertility were associated with less extreme manifestations of SPGF, such as SO.

## Lisbon clinical group co-authors and IVIRMA group co-authors

Ana Aguiar, (Unidade de Medicina da Reproducao, Hospital de Santa Maria, Centro Hospitalar Lisboa Norte, Lisboa, Portugal); Carlos Calhaz-Jorge, (Unidade de Medicina da Reproducao, Hospital de Santa Maria, Centro Hospitalar Lisboa Norte, Lisboa, Portugal); Joaquim Nunes, (Unidade de Medicina da Reproducao, Hospital de Santa Maria, Centro Hospitalar Lisboa Norte, Lisboa, Portugal); Sandra Sousa (Unidade de Medicina da Reproducao, Hospital de Santa Maria, Centro Hospitalar Lisboa Norte, Lisboa, Portugal), and Sónia Correia (Centro de Medicina Reprodutiva, Maternidade Alfredo da Costa, Centro Hospitalar Lisboa Central, Lisboa, Portugal); Maria Graça Pinto(Centro de Medicina Reprodutiva, Maternidade Alfredo da Costa, Centro Hospitalar Lisboa Central, Lisboa, Portugal). Alberto Pacheco, (IVIRMA Madrid, Spain); Cristina González, (IVIRMA Sevilla, Spain); Susana Gómez, (IVIRMA Lisboa, Portugal); David Amorós, (IVIRMA Barcelona, Spain); Jesús Aguilar, (IVIRMA Vigo, Spain); Fernando Quintana, (IVIRMA Bilbao, Spain).

## Data Availability

The data generated in this study are either contained in the article file and its Supplementary Material or available upon reasonable request to the corresponding author.

## References

[B1] Agarwal A. Baskaran S. Parekh N. Cho C. L. Henkel R. Vij S. (2021). Male infertility. Lancet 397, 319–333. 10.1016/S0140-6736(20)32667-2 33308486

[B2] Alves I. Houle A. A. Hussin J. G. Awadalla P. (2017). The impact of recombination on human mutation load and disease. Philos. Trans. R. Soc. Lond. B Biol. Sci. 372, 20160465. 10.1098/rstb.2016.0465 29109227 PMC5698626

[B3] Araujo T. F. Friedrich C. Grangeiro C. H. P. Martelli L. R. Grzesiuk J. D. Emich J. (2020). Sequence analysis of 37 candidate genes for male infertility: Challenges in variant assessment and validating genes. Andrology 8, 434–441. 10.1111/andr.12704 31479588

[B4] Aston K. I. Krausz C. Laface I. Ruiz-Castane E. Carrell D. T. (2010). Evaluation of 172 candidate polymorphisms for association with oligozoospermia or azoospermia in a large cohort of men of European descent. Hum. Reprod. 25, 1383–1397. 10.1093/humrep/deq081 20378615

[B5] Auton A. Brooks L. D. Durbin R. M. Garrison E. P. Kang H. M. Korbel J. O. (2015). A global reference for human genetic variation. Nature 526, 68–74. 10.1038/nature15393 26432245 PMC4750478

[B6] Barrett J. C. (2009). Haploview: Visualization and analysis of SNP genotype data. Cold Spring Harb. Protoc. 2009, pdb.ip71. pdb ip71. 10.1101/pdb.ip71 20147036

[B7] Bellil H. Ghieh F. Hermel E. Mandon-Pepin B. Vialard F. (2021). Human testis-expressed (TEX) genes: A review focused on spermatogenesis and male fertility. Basic Clin. Androl. 31, 9. 10.1186/s12610-021-00127-7 33882832 PMC8061069

[B8] Benjamini Y. Drai D. Elmer G. Kafkafi N. Golani I. (2001). Controlling the false discovery rate in behavior genetics research. Behav. Brain Res. 125, 279–284. 10.1016/s0166-4328(01)00297-2 11682119

[B9] Bjorndahl L. Kirkman Brown J. other Editorial Board Members of the WHO Laboratory Manual for the Examination and Processing of Human Semen (2022). The sixth edition of the WHO laboratory manual for the examination and processing of human semen: Ensuring quality and standardization in basic examination of human ejaculates. Fertil. Steril. 117, 246–251. 10.1016/j.fertnstert.2021.12.012 34986984

[B10] Cannarella R. Condorelli R. A. Duca Y. La Vignera S. Calogero A. E. (2019). New insights into the genetics of spermatogenic failure: A review of the literature. Hum. Genet. 138, 125–140. 10.1007/s00439-019-01974-1 30656449

[B11] Cannarella R. Condorelli R. A. Mongioi L. M. La Vignera S. Calogero A. E. (2020). Molecular Biology of spermatogenesis: Novel Targets of apparently idiopathic male infertility. Int. J. Mol. Sci. 21, 1728. 10.3390/ijms21051728 32138324 PMC7084762

[B12] Cannarella R. Condorelli R. A. Paolacci S. Barbagallo F. Guerri G. Bertelli M. (2021). Next-generation sequencing: Toward an increase in the diagnostic yield in patients with apparently idiopathic spermatogenic failure. Asian J. Androl. 23, 24–29. 10.4103/aja.aja_25_20 32655042 PMC7831827

[B13] Capilla L. Garcia Caldes M. Ruiz-Herrera A. (2016). Mammalian meiotic recombination: A toolbox for genome evolution. Cytogenet. Genome Res. 150, 1–16. 10.1159/000452822 27926907

[B14] Carithers L. J. Moore H. M. (2015). The genotype-tissue expression (GTEx) project. Biopreserv. Biobank. 13, 307–308. 10.1089/bio.2015.29031.hmm 26484569 PMC4692118

[B15] Carvalho-Silva D. Pierleoni A. Pignatelli M. Ong C. Fumis L. Karamanis N. (2019). Open Targets Platform: New developments and updates two years on. Nucleic Acids Res. 47, D1056–D1065. 10.1093/nar/gky1133 30462303 PMC6324073

[B16] Cervan-Martin M. Bossini-Castillo L. Rivera-Egea R. Garrido N. Lujan S. Romeu G. (2021). Effect and *in silico* characterization of genetic variants associated with severe spermatogenic disorders in a large Iberian cohort. Andrology 9, 1151–1165. 10.1111/andr.13009 33784440

[B17] Cervan-Martin M. Bossini-Castillo L. Rivera-Egea R. Garrido N. Lujan S. Romeu G. (2020a). Evaluation of male fertility-associated loci in a European population of patients with severe spermatogenic impairment. J. Pers. Med. 11, 22. 10.3390/jpm11010022 33383876 PMC7823507

[B18] Cervan-Martin M. Castilla J. A. Palomino-Morales R. J. Carmona F. D. (2020b). Genetic landscape of nonobstructive azoospermia and new perspectives for the clinic. J. Clin. Med. 9, 300. 10.3390/jcm9020300 31973052 PMC7074441

[B19] Cervan-Martin M. Suazo-Sanchez M. I. Rivera-Egea R. Garrido N. Lujan S. Romeu G. (2020c). Intronic variation of the SOHLH2 gene confers risk to male reproductive impairment. Fertil. Steril. 114, 398–406. 10.1016/j.fertnstert.2020.02.115 32690270

[B20] Chakravarthi V. P. Ghosh S. Roby K. F. Wolfe M. W. Rumi M. a. K. (2020). A gatekeeping role of ESR2 to maintain the primordial follicle reserve. Endocrinology 161, bqaa037. 10.1210/endocr/bqaa037 32141511

[B21] Chalmel F. Lardenois A. Evrard B. Mathieu R. Feig C. Demougin P. (2012). Global human tissue profiling and protein network analysis reveals distinct levels of transcriptional germline-specificity and identifies target genes for male infertility. Hum. Reprod. 27, 3233–3248. 10.1093/humrep/des301 22926843

[B22] Chang C. C. Chow C. C. Tellier L. C. Vattikuti S. Purcell S. M. Lee J. J. (2015). Second-generation PLINK: Rising to the challenge of larger and richer datasets. Gigascience 4, 7. 10.1186/s13742-015-0047-8 25722852 PMC4342193

[B23] Chen S. R. Hao X. X. Zhang Y. Deng S. L. Wang Z. P. Wang Y. Q. (2016). Androgen receptor in Sertoli cells regulates DNA double-strand break repair and chromosomal synapsis of spermatocytes partially through intercellular EGF-EGFR signaling. Oncotarget 7, 18722–18735. 10.18632/oncotarget.7916 26959739 PMC4951324

[B86] Cioppi F. Rosta V. Krausz C. (2021). Genetics of Azoospermia. Int. J. Mol. Sci. 22, 3264. 33806855 10.3390/ijms22063264 PMC8004677

[B24] Colombo R. Pontoglio A. Bini M. (2017). Two novel TEX15 mutations in a family with nonobstructive azoospermia. Gynecol. Obstet. Invest. 82, 283–286. 10.1159/000468934 28355598

[B25] Cooper T. G. Noonan E. Von Eckardstein S. Auger J. Baker H. W. Behre H. M. (2010). World Health Organization reference values for human semen characteristics. Hum. Reprod. Update 16, 231–245. 10.1093/humupd/dmp048 19934213

[B26] Cui H. Dhroso A. Johnson N. Korkin D. (2015). The variation game: Cracking complex genetic disorders with NGS and omics data. Methods 79-80, 18–31. 10.1016/j.ymeth.2015.04.018 25944472

[B27] Dahlet T. Truss M. Frede U. Al Adhami H. Bardet A. F. Dumas M. (2021). E2F6 initiates stable epigenetic silencing of germline genes during embryonic development. Nat. Commun. 12, 3582. 10.1038/s41467-021-23596-w 34117224 PMC8195999

[B28] Davis C. A. Hitz B. C. Sloan C. A. Chan E. T. Davidson J. M. Gabdank I. (2018). The encyclopedia of DNA elements (ENCODE): Data portal update. Nucleic Acids Res. 46, D794–D801. 10.1093/nar/gkx1081 29126249 PMC5753278

[B29] Djurovic J. Stamenkovic G. Todorovic J. Aleksic N. Stojkovic O. (2020). Polymorphisms and haplotypes in VDR gene are associated with female idiopathic infertility. Hum. Fertil. 23, 101–110. 10.1080/14647273.2018.1515503 30221569

[B30] Dong S. Boyle A. P. (2019). Predicting functional variants in enhancer and promoter elements using RegulomeDB. Hum. Mutat. 40, 1292–1298. 10.1002/humu.23791 31228310 PMC6744346

[B31] Eijpe M. Heyting C. Gross B. Jessberger R. (2000). Association of mammalian SMC1 and SMC3 proteins with meiotic chromosomes and synaptonemal complexes. J. Cell Sci. 113, 673–682. 10.1242/jcs.113.4.673 10652260

[B32] Fainberg J. Kashanian J. A. (2019). Recent advances in understanding and managing male infertility. F1000Res 8, 670. 10.12688/f1000research.17076.1 PMC6524745 31143441

[B33] Fan H. Y. Liu Z. Shimada M. Sterneck E. Johnson P. F. Hedrick S. M. (2009). MAPK3/1 (ERK1/2) in ovarian granulosa cells are essential for female fertility. Science 324, 938–941. 10.1126/science.1171396 19443782 PMC2847890

[B34] Ghadirkhomi E. Angaji S. A. Khosravi M. Mashayekh M. R. (2022). Correlation of novel single nucleotide polymorphisms ofUSP26, TEX15, and TNP2 genes with male infertility in north west of Iran. Int. J. Fertil. Steril. 16, 10–16. 10.22074/IJFS.2021.521138.1058 35103426 PMC8808250

[B35] Gonzalez-Marin C. Gosalvez J. Roy R. (2012). Types, causes, detection and repair of DNA fragmentation in animal and human sperm cells. Int. J. Mol. Sci. 13, 14026–14052. 10.3390/ijms131114026 23203048 PMC3509564

[B36] Guo J. Grow E. J. Mlcochova H. Maher G. J. Lindskog C. Nie X. (2018). The adult human testis transcriptional cell atlas. Cell Res. 28, 1141–1157. 10.1038/s41422-018-0099-2 30315278 PMC6274646

[B37] Guo J. Nie X. Giebler M. Mlcochova H. Wang Y. Grow E. J. (2020). The dynamic transcriptional cell atlas of testis development during human puberty. Cell Stem Cell 26, 262–276. 10.1016/j.stem.2019.12.005 31928944 PMC7298616

[B38] Hernandez-Hernandez A. Lilienthal I. Fukuda N. Galjart N. Hoog C. (2016). CTCF contributes in a critical way to spermatogenesis and male fertility. Sci. Rep. 6, 28355. 10.1038/srep28355 27345455 PMC4921845

[B39] Houston B. J. Riera-Escamilla A. Wyrwoll M. J. Salas-Huetos A. Xavier M. J. Nagirnaja L. (2021). A systematic review of the validated monogenic causes of human male infertility: 2020 update and a discussion of emerging gene-disease relationships. Hum. Reprod. Update 28, 15–29. 10.1093/humupd/dmab030 34498060 PMC8730311

[B40] James R. D. Schmiesing J. A. Peters A. H. Yokomori K. Disteche C. M. (2002). Differential association of SMC1alpha and SMC3 proteins with meiotic chromosomes in wild-type and SPO11-deficient male mice. Chromosome Res. 10, 549–560. 10.1023/a:1020910601858 12498344

[B41] Jarvi K. Lo K. Fischer A. Grantmyre J. Zini A. Chow V. (2010). CUA Guideline: The workup of azoospermic males. Can. Urol. Assoc. J. 4, 163–167. 10.5489/cuaj.10050 20514278 PMC2874589

[B42] Jorgez C. J. Wilken N. Addai J. B. Newberg J. Vangapandu H. V. Pastuszak A. W. (2015). Genomic and genetic variation in E2F transcription factor-1 in men with nonobstructive azoospermia. Fertil. Steril. 103, 44–52. 10.1016/j.fertnstert.2014.09.021 25439843 PMC4282601

[B43] Khristi V. Chakravarthi V. P. Singh P. Ghosh S. Pramanik A. Ratri A. (2018). ESR2 regulates granulosa cell genes essential for follicle maturation and ovulation. Mol. Cell. Endocrinol. 474, 214–226. 10.1016/j.mce.2018.03.012 29580824

[B44] Kim J. S. Chae J. H. Cheon Y. P. Kim C. G. (2016). Reciprocal localization of transcription factors YY1 and CP2c in spermatogonial stem cells and their putative roles during spermatogenesis. Acta Histochem. 118, 685–692. 10.1016/j.acthis.2016.08.005 27612612

[B45] Krausz C. Cioppi F. Riera-Escamilla A. (2018). Testing for genetic contributions to infertility: Potential clinical impact. Expert Rev. Mol. diagn. 18, 331–346. 10.1080/14737159.2018.1453358 29540081

[B46] Krausz C. Riera-Escamilla A. (2018). Genetics of male infertility. Nat. Rev. Urol. 15, 369–384. 10.1038/s41585-018-0003-3 29622783

[B47] Kumar S. Ambrosini G. Bucher P. (2017). SNP2TFBS - a database of regulatory SNPs affecting predicted transcription factor binding site affinity. Nucleic Acids Res. 45, D139–D144. 10.1093/nar/gkw1064 27899579 PMC5210548

[B48] Leduc F. Nkoma G. B. Boissonneault G. (2008). Spermiogenesis and DNA repair: A possible etiology of human infertility and genetic disorders. Syst. Biol. Reprod. Med. 54, 3–10. 10.1080/19396360701876823 18543861

[B49] Li Q. Pangas S. A. Jorgez C. J. Graff J. M. Weinstein M. Matzuk M. M. (2008). Redundant roles of SMAD2 and SMAD3 in ovarian granulosa cells *in vivo* . Mol. Cell. Biol. 28, 7001–7011. 10.1128/MCB.00732-08 18809571 PMC2593383

[B50] Luo Y. Hitz B. C. Gabdank I. Hilton J. A. Kagda M. S. Lam B. (2020). New developments on the Encyclopedia of DNA Elements (ENCODE) data portal. Nucleic Acids Res. 48, D882–D889. 10.1093/nar/gkz1062 31713622 PMC7061942

[B51] Machiela M. J. Chanock S. J. (2015). LDlink: A web-based application for exploring population-specific haplotype structure and linking correlated alleles of possible functional variants. Bioinformatics 31, 3555–3557. 10.1093/bioinformatics/btv402 26139635 PMC4626747

[B52] Maeda I. Okamura D. Tokitake Y. Ikeda M. Kawaguchi H. Mise N. (2013). Max is a repressor of germ cell-related gene expression in mouse embryonic stem cells. Nat. Commun. 4, 1754. 10.1038/ncomms2780 23612295

[B53] Man P. S. Wells T. Carter D. A. (2014). Cellular distribution of Egr1 transcription in the male rat pituitary gland. J. Mol. Endocrinol. 53, 271–280. 10.1530/JME-14-0158 25139489

[B54] Neto F. T. Bach P. V. Najari B. B. Li P. S. Goldstein M. (2016). Spermatogenesis in humans and its affecting factors. Semin. Cell Dev. Biol. 59, 10–26. 10.1016/j.semcdb.2016.04.009 27143445

[B55] Okutman O. Muller J. Baert Y. Serdarogullari M. Gultomruk M. Piton A. (2015). Exome sequencing reveals a nonsense mutation in TEX15 causing spermatogenic failure in a Turkish family. Hum. Mol. Genet. 24, 5581–5588. 10.1093/hmg/ddv290 26199321

[B56] Oscanoa J. Sivapalan L. Gadaleta E. Dayem Ullah A. Z. Lemoine N. R. Chelala C. (2020). SNPnexus: A web server for functional annotation of human genome sequence variation (2020 update). Nucleic Acids Res. 48, W185–W192. 10.1093/nar/gkaa420 32496546 PMC7319579

[B57] Papatheodorou I. Moreno P. Manning J. Fuentes A. M. George N. Fexova S. (2020). Expression atlas update: From tissues to single cells. Nucleic Acids Res. 48, D77–D83. 10.1093/nar/gkz947 31665515 PMC7145605

[B58] Plaseski T. Noveski P. Popeska Z. Efremov G. D. Plaseska-Karanfilska D. (2012). Association study of single-nucleotide polymorphisms in FASLG, JMJDIA, LOC203413, TEX15, BRDT, OR2W3, INSR, and TAS2R38 genes with male infertility. J. Androl. 33, 675–683. 10.2164/jandrol.111.013995 22016351

[B59] Pohlers M. Truss M. Frede U. Scholz A. Strehle M. Kuban R. J. (2005). A role for E2F6 in the restriction of male-germ-cell-specific gene expression. Curr. Biol. 15, 1051–1057. 10.1016/j.cub.2005.04.060 15936277

[B60] Ponomarenko M. P. Sharypova E. B. Drachkova I. A. Savinkova L. K. Chadaeva I. V. Rasskazov D. A. (2020). Candidate SNP-markers altering TBP binding affinity for promoters of the Y-linked genes CDY2A, SHOX, and ZFY are lowering many indexes of reproductive potential in men. Vavilovskii Zhurnal Genet. Sel. 24, 785–793. 10.18699/VJ20.674 PMC8094035 33959695

[B61] Rivero-Hinojosa S. Pugacheva E. M. Kang S. Mendez-Catala C. F. Kovalchuk A. L. Strunnikov A. V. (2021). The combined action of CTCF and its testis-specific paralog BORIS is essential for spermatogenesis. Nat. Commun. 12, 3846. 10.1038/s41467-021-24140-6 34158481 PMC8219828

[B62] Rocca M. S. Di Nisio A. Sabovic I. Ghezzi M. Foresta C. Ferlin A. (2019). E2F1 copy number variations contribute to spermatogenic impairment and cryptorchidism by increasing susceptibility to heat stress. Andrology 7, 251–256. 10.1111/andr.12583 30659775

[B63] Ruan J. He X. J. Du W. D. Chen G. Zhou Y. Xu S. (2012). Genetic variants in TEX15 gene conferred susceptibility to spermatogenic failure in the Chinese Han population. Reprod. Sci. 19, 1190–1196. 10.1177/1933719112446076 22581801

[B64] Salas-Huetos A. Aston K. I. (2021). Defining new genetic etiologies of male infertility: Progress and future prospects. Transl. Androl. Urol. 10, 1486–1498. 10.21037/tau.2020.03.43 33850783 PMC8039605

[B65] Schopp T. Zoch A. Berrens R. V. Auchynnikava T. Kabayama Y. Vasiliauskaite L. (2020). TEX15 is an essential executor of MIWI2-directed transposon DNA methylation and silencing. Nat. Commun. 11, 3739. 10.1038/s41467-020-17372-5 32719317 PMC7385494

[B66] Sharma A. Minhas S. Dhillo W. S. Jayasena C. N. (2021). Male infertility due to testicular disorders. J. Clin. Endocrinol. Metab. 106, e442–e459. 10.1210/clinem/dgaa781 33295608 PMC7823320

[B67] Singh K. Jaiswal D. (2011). Human male infertility: A complex multifactorial phenotype. Reprod. Sci. 18, 418–425. 10.1177/1933719111398148 21421900

[B68] Skol A. D. Scott L. J. Abecasis G. R. Boehnke M. (2006). Joint analysis is more efficient than replication-based analysis for two-stage genome-wide association studies. Nat. Genet. 38, 209–213. 10.1038/ng1706 16415888

[B69] Szklarczyk D. Gable A. L. Lyon D. Junge A. Wyder S. Huerta-Cepas J. (2019). STRING v11: Protein-protein association networks with increased coverage, supporting functional discovery in genome-wide experimental datasets. Nucleic Acids Res. 47, D607–D613. 10.1093/nar/gky1131 30476243 PMC6323986

[B70] Teng Y. Wang Y. Fu J. Cheng X. Miao S. Wang L. (2011). Cyclin T2: A novel miR-15a target gene involved in early spermatogenesis. FEBS Lett. 585, 2493–2500. 10.1016/j.febslet.2011.06.031 21740905

[B71] Tian R. H. Huang Y. H. Chen H. X. Li P. Zhi E. L. Yao C. C. (2021). Successful microsurgical vasoepididymostomy for a case of cryptozoospermia. Asian J. Androl. 24, 436–437. 10.4103/aja202178 PMC9295479 34916476

[B72] Tomic D. Miller K. P. Kenny H. A. Woodruff T. K. Hoyer P. Flaws J. A. (2004). Ovarian follicle development requires Smad3. Mol. Endocrinol. 18, 2224–2240. 10.1210/me.2003-0414 15192076

[B73] Tourtellotte W. G. Nagarajan R. Bartke A. Milbrandt J. (2000). Functional compensation by Egr4 in Egr1-dependent luteinizing hormone regulation and Leydig cell steroidogenesis. Mol. Cell. Biol. 20, 5261–5268. 10.1128/mcb.20.14.5261-5268.2000 10866682 PMC85975

[B74] Wang X. Jin H. R. Cui Y. Q. Chen J. Sha Y. W. Gao Z. L. (2018). Case study of a patient with cryptozoospermia associated with a recessive TEX15 nonsense mutation. Asian J. Androl. 20, 101–102. 10.4103/1008-682X.194998 28303806 PMC5753545

[B75] Ward L. D. Kellis M. (2016). HaploReg v4: Systematic mining of putative causal variants, cell types, regulators and target genes for human complex traits and disease. Nucleic Acids Res. 44, D877–D881. 10.1093/nar/gkv1340 26657631 PMC4702929

[B76] Wu S. Hu Y. C. Liu H. Shi Y. (2009). Loss of YY1 impacts the heterochromatic state and meiotic double-strand breaks during mouse spermatogenesis. Mol. Cell. Biol. 29, 6245–6256. 10.1128/MCB.00679-09 19786570 PMC2786691

[B77] Wu J. Li D. Liu X. Li Q. He X. Wei J. (2021). Iddb: A comprehensive resource featuring genes, variants and characteristics associated with infertility. Nucleic Acids Res. 49, D1218–D1224. 10.1093/nar/gkaa753 32941628 PMC7779019

[B78] Yang F. Eckardt S. Leu N. A. Mclaughlin K. J. Wang P. J. (2008). Mouse TEX15 is essential for DNA double-strand break repair and chromosomal synapsis during male meiosis. J. Cell Biol. 180, 673–679. 10.1083/jcb.200709057 18283110 PMC2265566

[B79] Yang F. Lan Y. Pandey R. R. Homolka D. Berger S. L. Pillai R. S. (2020). TEX15 associates with MILI and silences transposable elements in male germ cells. Genes. Dev. 34, 745–750. 10.1101/gad.335489.119 32381626 PMC7263141

[B80] Zhang X. Ding M. Ding X. Li T. Chen H. (2015). Six polymorphisms in genes involved in DNA double-strand break repair and chromosome synapsis: Association with male infertility. Syst. Biol. Reprod. Med. 61, 187–193. 10.3109/19396368.2015.1027014 26086992

