# Supplementary material for: Contribution of TEX15 genetic variants to the risk of developing severe non-obstructive oligozoospermia
Source: Front Cell Dev Biol. 2022 Dec 15;10:1089782. doi: 10.3389/fcell.2022.1089782 (PMC9797780; doi:10.3389/fcell.2022.1089782)
Supplement: Supplementary file 1 [file DataSheet1.PDF]

Frontiers in Cell and Developmental Biology

Supplementary Figures

Contribution of TEX15 genetic variants to the risk of developing severe oligozoospermia

Andrea Guzmán-Jiménez[1,2,*], Sara González-Muñoz[1,2,*], Miriam Cerván-Martín[1,2], Rocío Rivera-Egea[3,4], Nicolás Garrido [4,5], Saturnino Luján [5], Samuel Santos-Ribeiro[6,7], IVIRMA Group[8], Lisbon Clinical Group[8], José A. Castilla[2,9,10], M. Carmen Gonzalvo[2,9], Ana Clavero[2,9], F. Javier Vicente[2,11], Vicente Maldonado[12], Javier Villegas-Salmerón[1], Miguel Burgos[1], Rafael Jiménez[1], Maria Graça Pinto[13], Isabel Pereira[14], Joaquim Nunes[14], Josvany Sánchez-Curbelo[15], Olga López-Rodrigo[15], Iris Pereira-Caetano[16], Patricia Isabel Marques[17,18], Filipa Carvalho[19], Alberto Barros[19], Lluís Bassas[15], Susana Seixas[17,18], João Gonçalves[18,20], Alexandra M. Lopes[17,18], Sara Larriba[21], Rogelio J. Palomino-Morales[2,22], F. David Carmona[1,2,¶], Lara Bossini-Castillo[1,2,¶].

* These authors share first authorship.
¶ These authors share last authorship.

Supplementary Figure S1. Gene expression of *TEX15* in different tissues and cell types of the testis. **A)** The upper panel shows the expression of the *TEX15* gene in transcripts per million (TPM) according to the GTEx project (data source: GTEx Analysis Release V8 [1]). Gene expression in the testis is highlighted with a blue box. The violin plots illustrate the density distribution and the median values are shown by the white line. **B)** The lower panel represents the dimension reduction (t-SNE) plots of single-cell transcriptome data in **B1)** puberty and **B2)** young adult human testes based on the RNA-seq dataset included in the Human Testis Atlas browser by Cairns Lab @Utah [2,3]. Single cells are represented as coloured dots and the different colours indicate cluster identities. Specific expression patterns of *TEX15* projected on the t-SNE plot is shown. Related to Young Adult Atlas cell type, 1: SSCs, 2: Differentiating spermatogonia, 3: Early primary spermatocytes, 4: Late primary spermatocytes, 5: Round spermatids, 6: Elongated spermatids, 7: Sperm, 8: Sperm. 9: Macrophages, 10: Endothelial Cells, 11: Myoid Cells, 12: Sertoli Cells, 13: Leydig Cells [2,3].

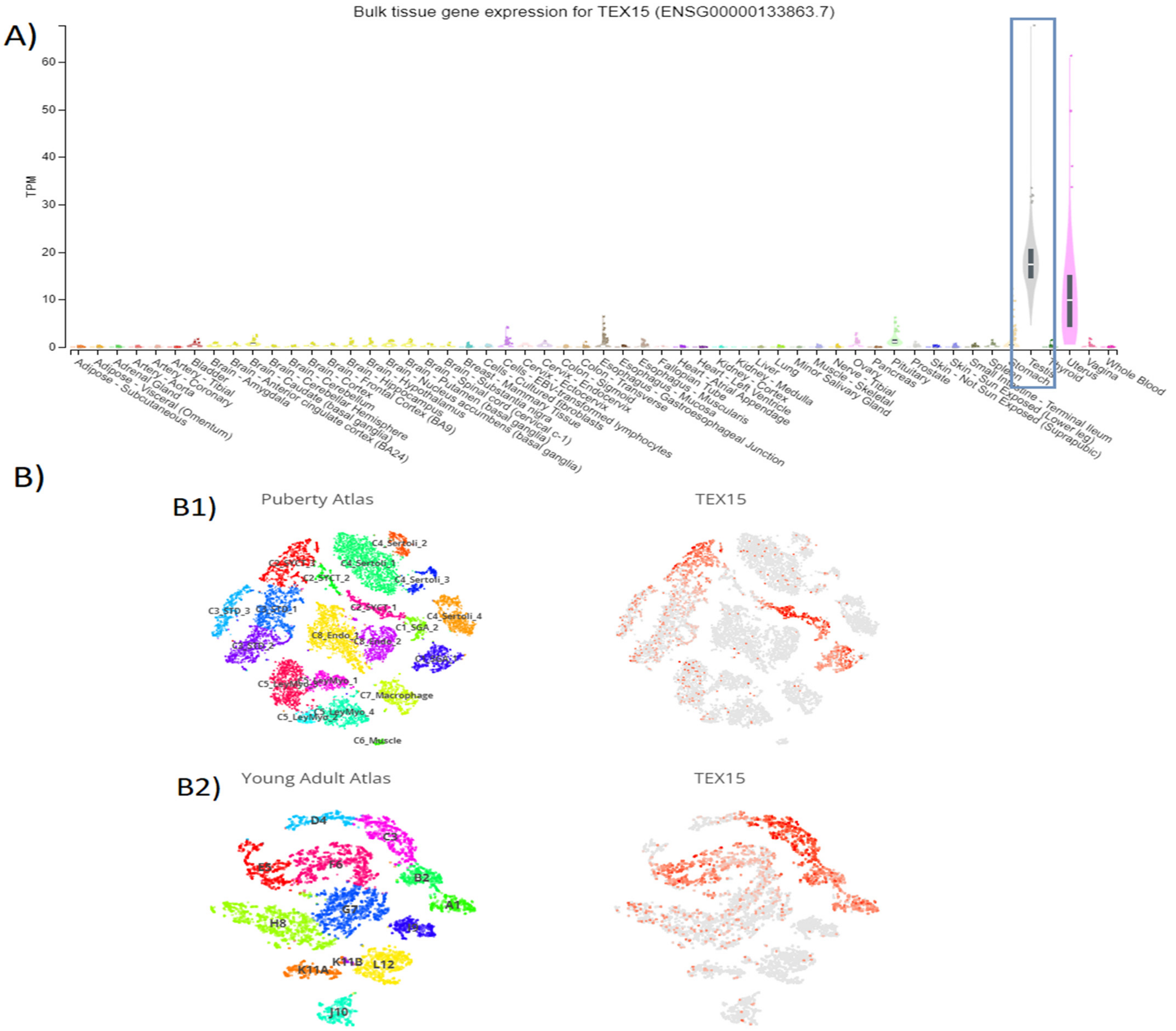

Supplementary Figure S2. Scatter plots for *TEX15*-rs323346 and its linked variants, according the African (AFR), East Asian (EAS), European (EUR) and South Asian population data of the 1000 genome project phase III [4]. The tagger variant is marked with a triangle. The Y-axis indicates the R^2 values with rs323346 and the X-axis the chromosome positions in base pairs (BP). MAF, minor allele frequency.

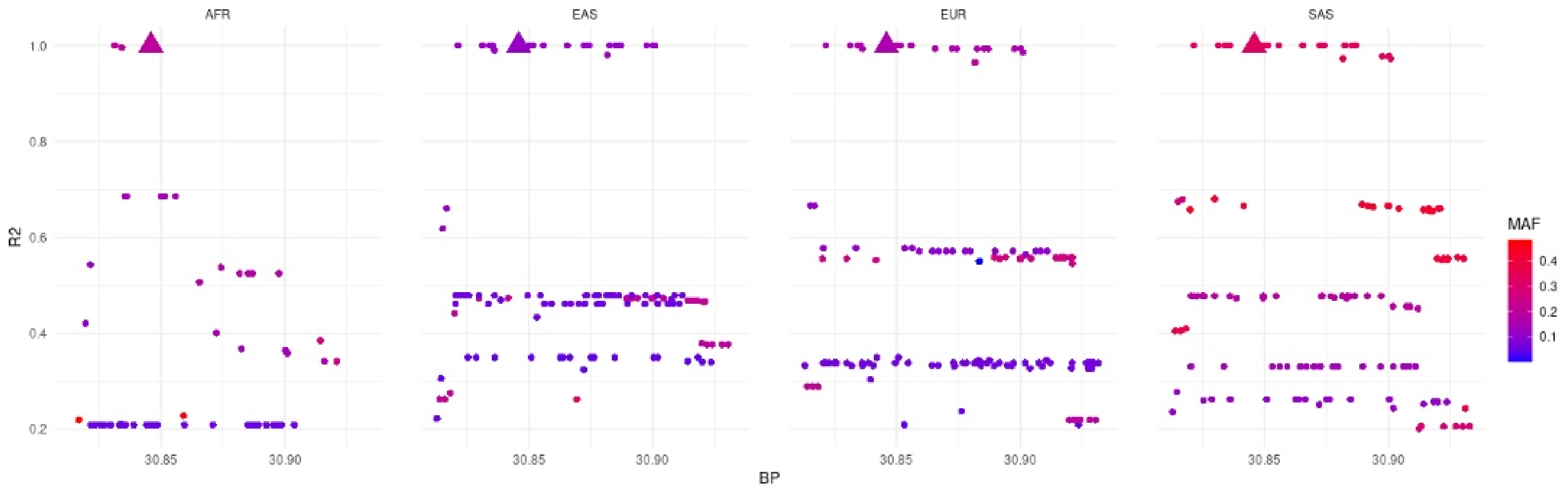

REFERENCES

1. Carithers LJ, Moore HM. The Genotype-Tissue Expression (GTEx) Project. Biopreservation and biobanking. 2015; 13(5):307-308.

2. Guo J, Nie X, Giebler M, Mlcochova H, Wang Y, Grow EJ, et al. The Dynamic Transcriptional Cell Atlas of Testis Development during Human Puberty. Cell stem cell. 2020; 26(2):262-276 e264.

3. Guo J, Grow EJ, Mlcochova H, Maher GJ, Lindskog C, Nie X, et al. The adult human testis transcriptional cell atlas. Cell research. 2018; 28(12):1141-1157.

4. Auton A, Brooks LD, Durbin RM, Garrison EP, Kang HM, Korbel JO, et al. A global reference for human genetic variation. Nature. 2015; 526(7571):68-74.
